# The Impact of Hospitality on Air Quality at a Major Sporting Event

**DOI:** 10.1021/acsestair.5c00142

**Published:** 2026-02-02

**Authors:** W. Joe F. Acton, Vipul Lalchandani, Mao Du, Siqi Hou, Deepchandra Srivastava, Zongbo Shi, William J. Bloss

**Affiliations:** School of Geography, Earth and Environmental Sciences, 1724 University of Birmingham, Birmingham B15 2TT, U.K.

**Keywords:** air quality, Commonwealth Games, particulate matter, cooking emissions, PMF

## Abstract

Large scale sporting and cultural events attract many spectators to a single site, leading to changed emissions and potentially creating local air pollution hot spots. Here, we monitored the air quality during the Birmingham 2022 Commonwealth Games, held from July 28th to August 8th, 2022, with 323,000 spectators attending the athletics events, including during the opening and closing ceremonies at the (open air) Alexander Stadium in Birmingham, UK. Particulate (PM_2.5_ and PM_10_) concentrations in fan areas around the stadium peaked ahead of the athletics events and opening and closing ceremonies with PM_2.5_ concentrations up to 10 times higher than at nearby urban background monitoring stations. For a spectator attending a full day of events at Alexander Stadium, this would represent a 125% increase in their exposure to PM_2.5_ relative to the urban background. Nonrefractory particulate composition in these periods was dominated by organics. Four factors were identified from Positive Matrix Factorization (PMF) analysis of particle composition data recorded using a Quadrupole Aerosol Chemical Speciation Monitor (Q-ACSM): two representing cooking aerosol accounting for 71% of the total PM mass during the athletic sessions demonstrating that cooking sources were responsible for the majority of particulate pollution at the venue. The high particulate concentrations at this venue were driven by fast food production at temporary concession stands, common across many large events, leading to a large increase in particulate matter exposure for staff and visitors.

## Graphical abstract

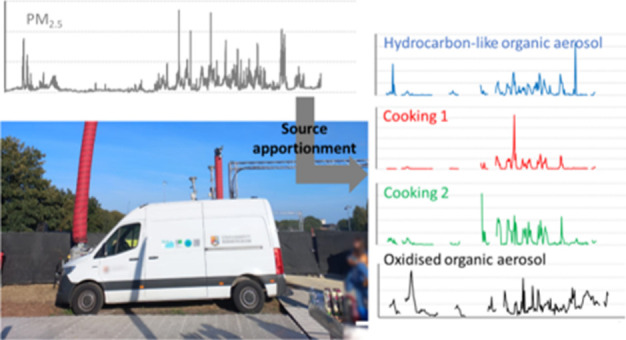

## Introduction

Globally, air pollution causes over 6.5 million deaths a year.^1^ In the United Kingdom (UK), air pollution is the largest environmental threat to human health with up to 29,000–43,000 premature deaths a year attributable to long-term exposure to outdoor air pollution.^2^ In urban areas in the UK, PM_2.5_ (particles with a diameter of less than 2.5 μm) are the pollutants with the greatest impact on human health and the economy.^3^ PM_2.5_ together with nitrogen oxides (NO_*x*_) are the focus of most national and regional mitigation strategies.

Major sporting and cultural events such as music festivals and the Olympic and Commonwealth Games attract thousands of spectators to cities and festival grounds, contributing significantly to regional economies and providing cultural and social enrichment. In 2019, UK music festivals attracted over 5.2 million people,^4^ and the Birmingham 2022 Commonwealth Games attracted over 1.5 million spectators.^5^

Air quality at these large sporting and cultural events is heavily dependent upon local meteorology and emission sources in the surrounding region, as well as emissions directly associated with the event. The air quality in cities hosting major sporting events has been extensively reported, including the Olympic Games and Commonwealth Games in Rio de Janeiro,^6–10^ London,^11^ Delhi,^12–15^ and Beijing.^16–19^ These studies focus on the ambient air quality in the surrounding city during the Games and, in the case of Beijing and Delhi, the effect of interventions to improve regional air quality.^12,16,18^

There have, however, been fewer studies focusing on air quality at large event venues and how this differs from the surrounding city. The air quality at these sites is impacted by a large number of local sources including spectator transport, catering, on site generators, and fireworks. Bisht et al.^20^ assessed the air quality at ten locations across Delhi before and during the 2010 Commonwealth Games, finding that concentrations of PM_2.5_ were ∼18% lower inside the stadium than outside. This was attributed to unfavorable metrological conditions for the dispersal of PM_2.5_. Particulate sources within a football stadium were investigated by Faber et al.,^21^ who found a strongly elevated mass concentration of organic aerosols associated with cigarette smoking and cooking.

The large number of spectators visiting these sites gives rise to a significant impact on collective short-term exposure to air pollution. For staff, volunteers, and athletes who attend multiple events, this results in increased long-term exposure. The air quality at sporting venues has also been shown to impact athletic performance. Hodgson et al.^22^ investigated the impact of air quality on the performance of 5-km athletes at Diamond League events, showing that ozone and particulate matter reduced performance.

Large sporting and cultural events commonly provide a broad range of mobile catering stands. Cooking has been shown to make a significant contribution to organic aerosol concentrations in urban environments.^23,24^ Cooking activities at temporary on-site catering venues are, therefore, a potential source of particulate emissions on site. Fireworks are associated with pollution spikes around major events. There have been several studies focusing on the impact of fireworks on particulate concentrations, with much of the research focusing on the Diwali festival in India^25^ and Lunar New Year celebrations in China.^26,27^ Singh et al.^28^ reviewed the impact of fireworks on particulate matter concentrations, finding that pollutant concentrations during firework events could peak at 2–8 times above local ambient concentrations.

The Birmingham 2022 Commonwealth Games (B2022) were held from July 28th to August 8th 2022 across 14 venues in the UK West Midlands region and one venue in London. The Games attracted 6600 athletes and team officials and over 1.5 million visitors to the region,^29^ with 323,000 ticketed spectators attending the athletics events at Alexander Stadium in Birmingham.^5^ This study aims to record air quality and identify pollutant sources in the fan areas adjacent to but outside Alexander Stadium, where spectators gathered before and between athletics sessions. These areas contained many catering and retail stands, typical of those at other large sport events or music festivals. Given the high number of attendees, environmental conditions in these areas may have a significant impact on the short-term exposure of a large population.

## Methods

### Site Description

Alexander Stadium is an open air athletics stadium with an 18,000-spectator capacity (increased to 32,000 during the B2022 Games period) located in the Perry Barr Region of Birmingham, UK (Figure 1). It hosted the B2022 athletics events, opening ceremony, and closing ceremony. The stadium is surrounded by residential properties to the north and west, an area of allotments, the A34 main road to the south, and the M6 motorway to the northeast.

**Figure 1:**
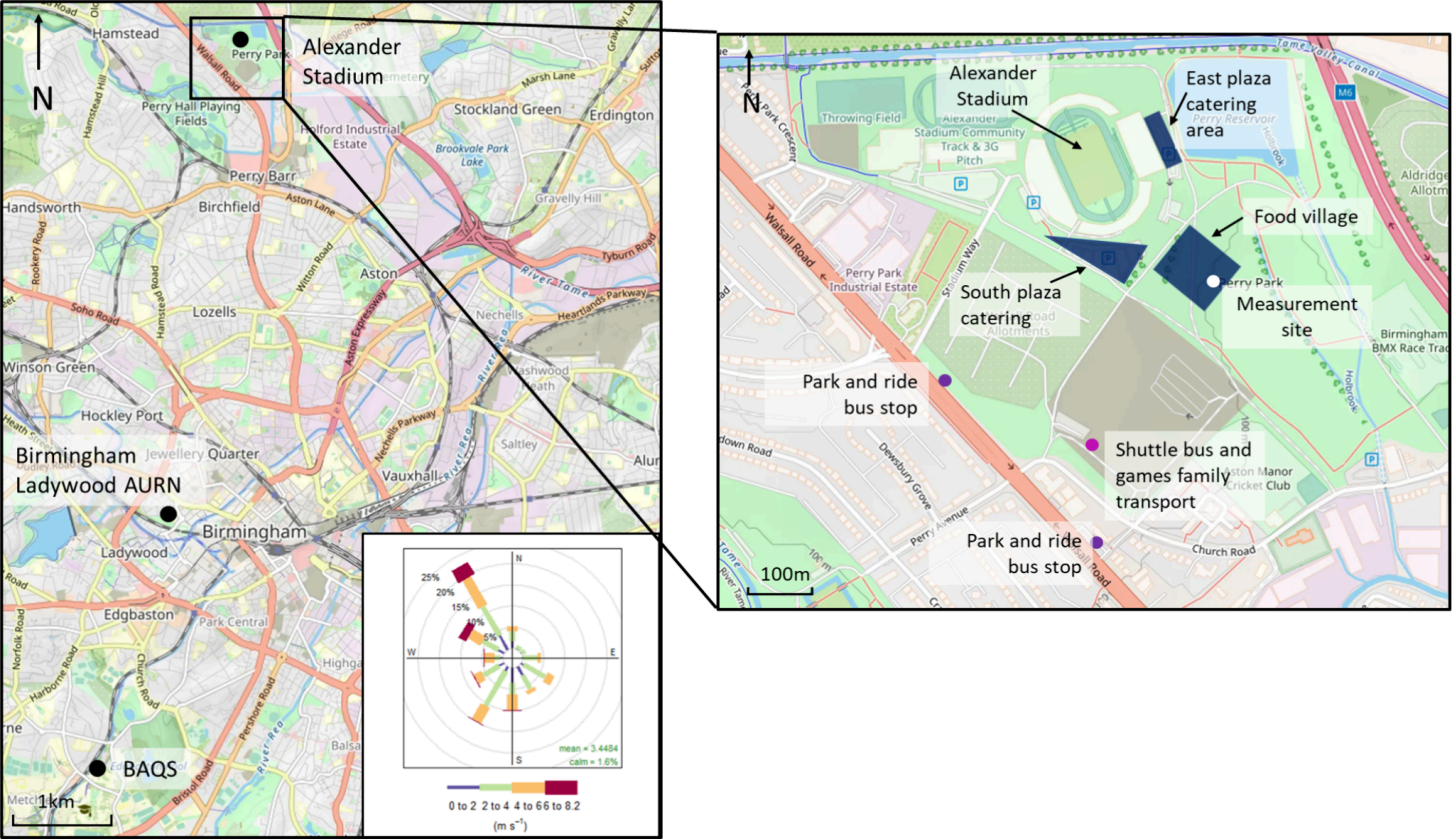
Location of the measurement site at Alexander Stadium and the Birmingham Ladywood and BAQS background monitoring sites (black). The expanded section shows Alexander Stadium, the measurement site, main catering areas, and the spectator park and ride (purple) and shuttle bus (pink) drop off locations. Wind rose shows Games period wind direction and speed recorded at BAQS (Figure S3). Map data were obtained from OpenStreetMap under the Open Database License.

Air quality instruments were installed in an air-conditioned Mercedes e-sprinter van sited in a fan zone adjacent to Alexander Stadium (52° 31′ 47.59″ N, 1° 54′ 11.26′′ W, measurement site in Figure 1). The instruments sampled from the rood of the van at a height of ∼3 m. The Games period ran from 28th July to 8th August 2022, and air quality measurements started from 19th July. During the Games, vehicles were prevented from accessing the Alexander stadium site. Spectator shuttle buses ran from the city center and park-and-ride sites, stopping along and near the A34 to the south of the stadium. Three fan zones around the stadium (Figure, 1) hosted multiple concession stands offering a wide range of hot food. Alongside grid supply, power was generated on site using hydrotreated vegetable oil fuelled generators.

### Instrument Setup

Concentrations of PM_1_, PM_2.5_, PM_4_, and PM_10_ were measured at 1 min resolution using a Fidas 200 optical particle counter (Palas, Germany) sampling from an inlet on the roof of the van. The mass concentrations and chemical composition of PM_2.5_ were recorded in real-time 30 min resolution using a Quadrupole Aerosol Chemical Speciation Monitor equipped with a PM_2.5_ lens and a capture vaporizer (Q-ACSM; Aerodyne Research, USA). The detailed methodology and operation of Q-ACSM can be found elsewhere.^30,31^ The mass concentrations of nonrefractory (NR) PM_2.5_ species including organics (Org), nitrate (NO_3_), ammonium (NH_4_), sulfate (SO_4_), and chloride (Chl) were analyzed using the standard TOF-ACSM data analysis software (Tofware v2.5.13) within Igor Pro (Wave-Metrics, Inc., Oregon USA). The Q-ACSM was calibrated for ionization efficiency following standard methodology with size-selected ammonium nitrate (NH_4_NO_3_) and ammonium sulfate ((NH_4_)_2_SO_4_) particles (300 nm) using a DMA-CPC setup.^32^ The relative ionization efficiencies for NH_4_ and SO_4_ were determined to be 6.94 and 1.33, respectively, while for organics, chloride, and nitrate, a standard RIE of 1.4, 1.4, and 1.1 were assumed. The detection limits for the species, determined from zero-filter measurements, were as follows: Org (0.68 μg m^–3^), NO_3_ (0.04 μg m^–3^), SO_4_ (0.12 μg m^–3^), NH_4_ (0.40 μg m^–3^), and Cl (0.11 μg m^–3^). The total mass of PM_2.5_ recorded using Q-ACSM was 95% of the mass measured using the Fidas for the athletics events period when both instruments were running simultaneously. The Fidas and Q-ACSM data sets had an *R* ^2^ of 0.51 (Figure S1). This moderate correlation is likely a result of the Q-ACSM only capturing NR-PM_2.5_ and under recovering refractory mass, while Fidas provides a measure of total PM_2.5_. During some peaks in PM_2.5_, the concentration measured using Q-ACSM exceeded that recorded using the Fidas. However, the difference in these measurements remained within the measurement uncertainty. The Fidas has an uncertainty of 9.7% for PM_2.5_, and the uncertainty in Q-ACSM measurements has been shown to be 9, 15, 19, 28, and 36% for nitrate, organic matter, sulfate, and ammonium, respectively.^33^

The elemental composition of particles was monitored using an Xact 625i (SailBri Cooper Inc., USA), which uses nondestructive energy dispersive X-ray fluorescence analysis to determine elemental concentrations collected on a filter tape. The Xact ran with a 1-h sampling time and a flow rate of 16.7 lpm, during which particulates were collected onto a sampling tape and then analyzed using X-ray fluorescence (XRF), with the instrument switching between PM_2.5_ and PM_10_. Hourly samples were recorded from 30th to 31st July and from 3rd to 6th August, with 22 elements detected. Fifteen elements contributed significantly to the total measured elemental composition: S, Ca, Fe, K, Te, Cl, Pd, Zn, Sb, Cu, Sn, Ti, Br, Ba, and Pb. Al and Si were excluded from analysis, as the signals were not considered reliable. This is consistent with previous studies that have reported errors in XRF measurements of these elements.^34,35^ Sampling and analysis are performed continuously with automatic energy alignment checks (using Cr and Nb rods) and automatic QA upscale (using Cr, Pb, Cd, and Nb rods) performed for 15 min (for Cr and Nb) at midnight each day. XRF calibration was performed using thin film standards of Ca, Mn, Zn, Pb, and Cd; the results were within the limit of ±5%, and the flow and leak checks were done before and after the campaign.

Ultrafine particles were measured using a scanning mobility particle sizer (SMPS+C, Grimm Aerosoltechnik, Germany). The SMPS was employed to measure the total number concentration and the number size distribution of aerosol particles. This system comprises a differential mobility analyzer (DMA) and a condensation particle counter (CPC) model 3776 (TSI). The CPC utilized butanol as the working fluid and is capable of measuring particle concentrations up to 3 × 10^5^ particles cm^–3^. For particle number size distribution measurements, the medium U-DMA evaluates particle sizes in the range of 5.5 to 350.4 nm. The system’s inlet was installed on the roof of the van, positioned approximately 1.5 m from the PM_2.5_ cutting head to the SMPS instrument. Data from the SMPS were recorded at a temporal resolution of 5 min, and the sample flow rates were consistently maintained at 1.5 L min^–1^. To ensure laminar flow conditions within the samples, a sheath-to-sample flow rate ratio of 10:1 was employed. The SMPS system was equipped with the standard Grimm 241Am neutralizer (model 5.521), which possesses an initial activity of 3.7 MBq, effectively neutralizing electrostatic charges on the aerosol particles.

NO and NO_2_ were measured using a Thermo Fisher 42i gas analyzer (Thermo Fisher, USA) sampling from an inlet installed on the roof of the van. Meteorological data were processed using the R openair package.^36^

### Supporting Measurements

Meteorology and supplementary air quality measurements were made at two nearby (5–9 km) locations, unaffected by Commonwealth Games events, for comparison with those obtained from the Alexander Stadium. The Birmingham Air Quality Supersite (BAQS), an urban background site on the University of Birmingham campus in Edgbaston (52° 27′ 19.71” N, 1° 55′ 44.35” W) 8.6 km Southwest of Alexander Stadium. Air quality data were also taken from the DEFRA urban background monitoring station at Birmingham Ladywood in central Birmingham (52° 28′ 52.85’’ N, 1° 55′ 5.65’’ W) 5.6 km Southwest of Alexander Stadium.

Back trajectories were calculated using the HYSPLIT-WEB Trajectory Model.^37,38^ Back trajectories were run for 24 h with an arrival altitude of 2 m and meteorology from the GDAS1 archive.

### Source Apportionment

The particle composition data provided by the Q-ACSM was analyzed using positive matrix factorization (PMF)^39^ to investigate the aerosol sources. The Q-ACSM mass spectral matrix was processed using the PMF Evaluation Tool Software v3.08 described by Ulbrich et al.^40^ Following the method described by Ulbrich et al.^40^ and applied by Sun et al.,^41^ organic ion fragments in the mass range of 12–98 amu were included in the matrix. The weak variables with signal-to noise (S/N) ratios <0.2 were removed and S/N < 2 down-weighted. Ions at *m*/*z* = 16, 17, 18, and 28, which are scaled to the organic *m*/*z* 44 signal, were excluded from the PMF and recalculated after the analysis.

PMF was run from 2-factor to 8-factor solution. Q/Q exp showed a significant decrease until the 4 factor solution, as shown in Figure S6. In the 3-factor solution, two cooking related factors (COA-1 and COA-2) and an oxygenated organic aerosol factor (OOA) were identified. In the 4-factor solution, a clear hydrocarbon-like organic aerosol (HOA) factor was resolved with a minor contribution to the total OA mass (∼9%). The COA-2 factor in the solution has some mixing of oxygenated ions at *m*/*z* 18 and 44; however, the diurnal profile, high *m*/*z* 55 signal, and the presence of aromatic and unsaturated hydrocarbons indicate it to be associated with cooking organic aerosols. Therefore, the 4-factor solution was chosen.

## Results

### Meteorology

Weather conditions in Birmingham over the summer of 2022 were dominated by periods of high pressure, leading to a series of heat waves, defined here as periods in which the UK Health Security Agency issued a heat health alert. The B2022 Games fell between the July (17th–19th) and August (9th–15th) heat waves with an average temperature of 18.4 °C. Temperatures peaked at 27.0 °C at 16:00 (BST) on 8th August and the minimum temperature of 7.0 °C was observed at 4:00 on 6th August (Figure S2). There was little precipitation during the Games period, with showers in the morning of July 31st and light rain showers on August 2nd and 3rd. Wind speed was low throughout the Games period, averaging 3.4 m s^–1^.

Back trajectory analysis using the HYSPLIT transport and dispersion model showed that air masses during the period in which athletics events were held at Alexander Stadium arrived from rural areas to the west of the Birmingham (Figure S4). Early in the event period (1st August) and at the end of this period (5th–7th August), these air masses originated in the North Atlantic, passing over Northern Ireland before reaching Birmingham. From 2nd to 4th August, these air masses originated in the southeast of Ireland, passing over southern Wales before reaching Birmingham.

### Particulate Concentrations

Particulate concentrations prior to the Games period averaged 16.0 (with a standard deviation of 25.0) and 6.1 (±5.0) μg m^–3^ for PM_10_ and PM_2.5_, respectively, with concentrations of PM_2.5_ spiking to over 70 μg m^–3^ on 27th July in the run-up to the opening ceremony (Figures 2 and 3a). During these periods, the PM_2.5_/PM_10_ ratio was low (0.39), indicating that these peaks are dominated by coarse particles. These spikes were likely caused by resuspended dust and exhaust fumes generated by vehicles operating near the measurement station during the final period of preparations on site before the Games.

**Figure 2:**
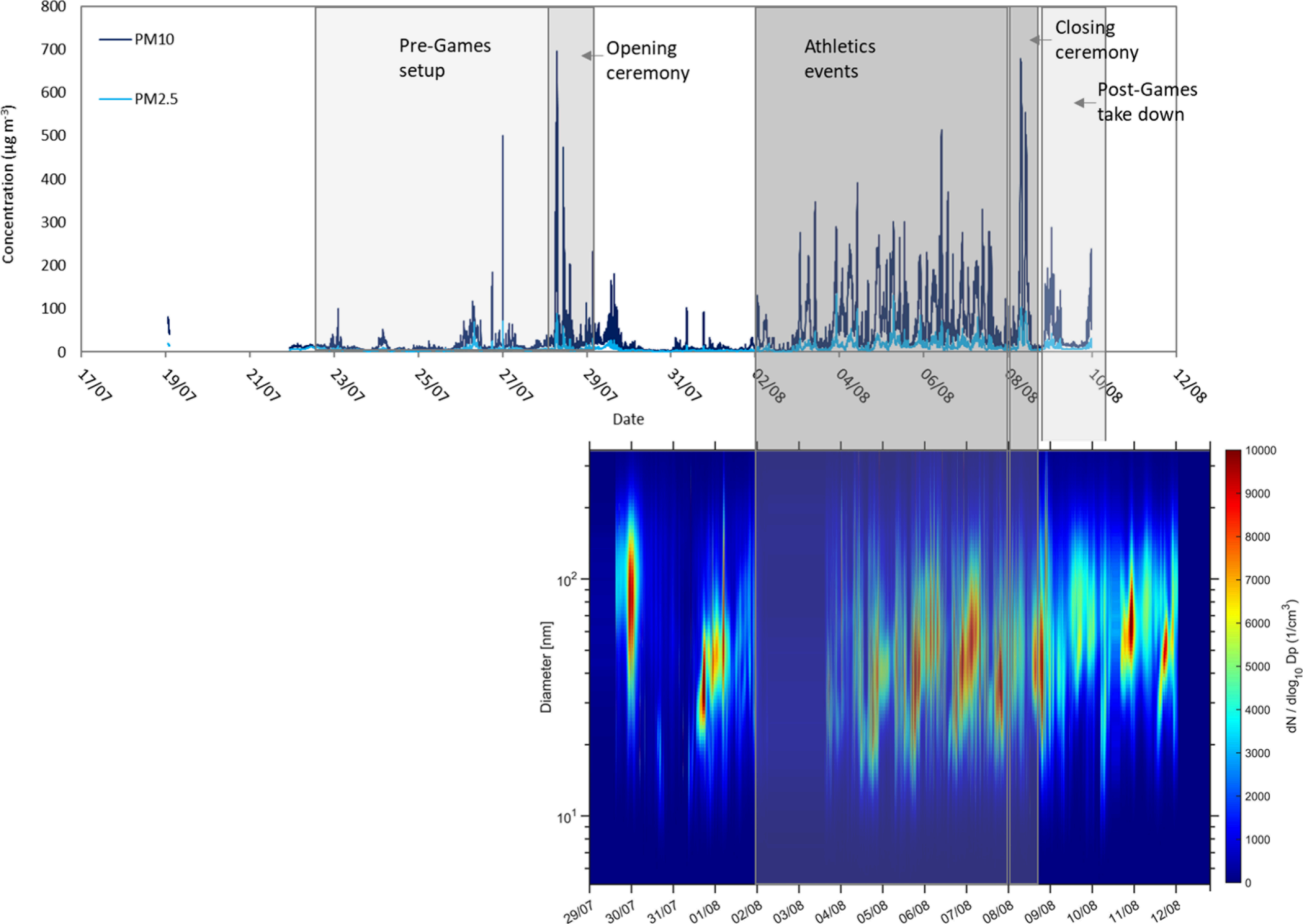
Top: time series of PM_10_ and PM_2.5_ concentrations at Alexander Stadium (1 min resolution) during the Commonwealth Games period. Bottom: time series of ultrafine particle number size distribution.

**Figure 3:**
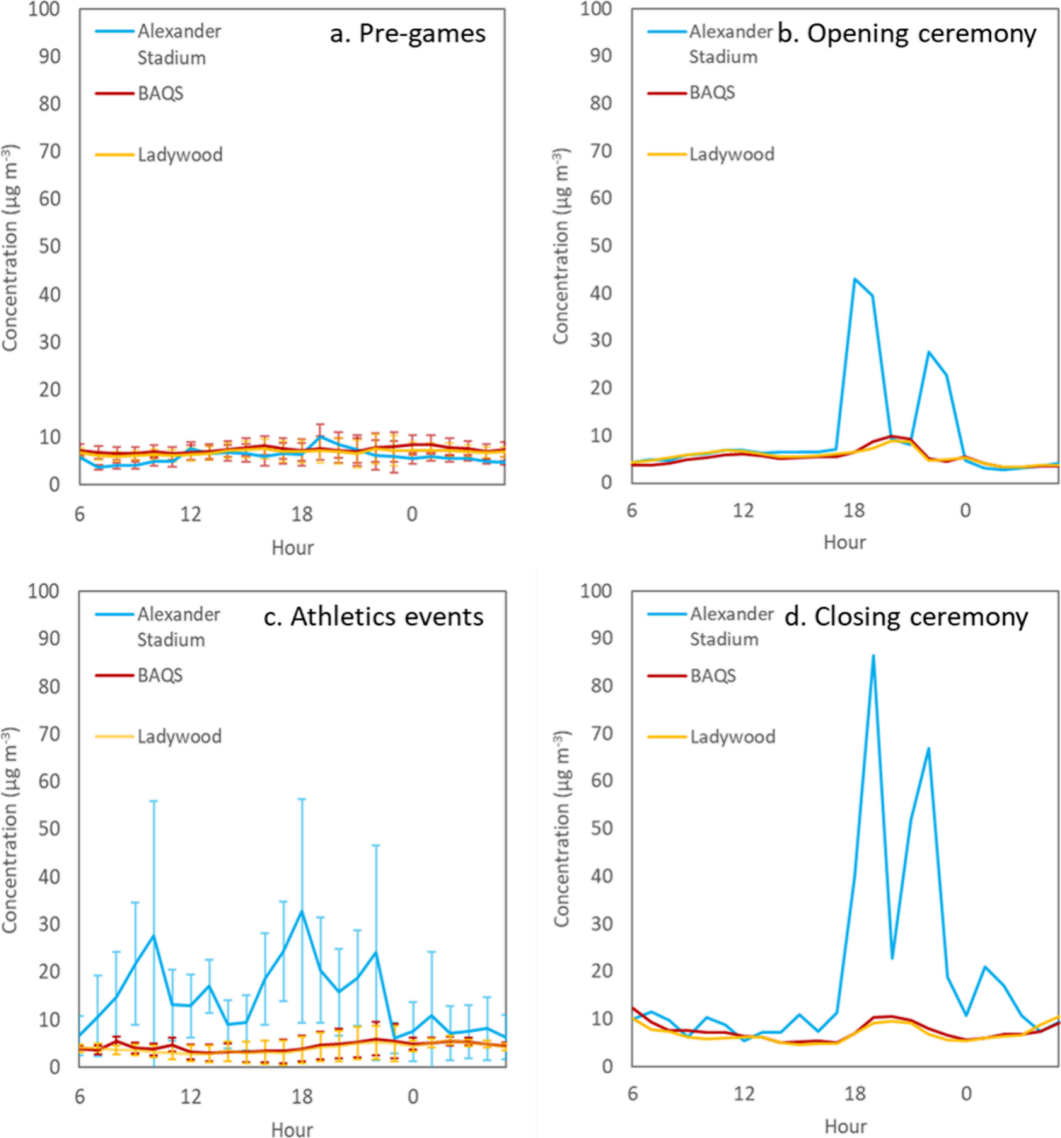
Diurnal cycle (note 6 am–6 am) of PM_2.5_ concentrations at Alexander Stadium (blue), BAQS (dark red), and Ladywood (orange) during the pre-Games period (19th–27th July), opening ceremony (28th July), athletics events (2nd–7th August), and closing ceremony (8th August). Error bars show 1 standard deviation.

Conditions at the stadium prior to the Games were comparable with those at urban background measurement stations in Birmingham, suggesting no significant influences from the surrounding roads. Average concentrations of PM_2.5_ at BAQS and Birmingham Ladywood in the pre-Games period were comparable to those observed at Alexander Stadium (pregames), with 7.4 (±5.1) and 6.9 (±4.4) μg m^–3^ recorded at BAQS and Birmingham Ladywood, respectively. The PM_2.5_/PM_10_ ratio in this period at Ladywood was 0.57, higher than that at Alexander Stadium.

The Commonwealth Games opening ceremony was held on the 28th July at Alexander Stadium. The ceremony ran from 20:00 to 22:30 and featured a large fireworks display, as well as extensive catering for spectators in the fan zones surrounding the stadium. PM_10_ and PM_2.5_ concentrations on the 28th July peaked ahead of the opening ceremony at 696.1 and 88.1 μg m^–3^, respectively, corresponding with resuspension of mineral dust generated by spectators arriving on site and cooking activities ahead of the Games. Average concentrations of PM_10_ and PM_2.5_ across the evening of the opening ceremony were 173.7 (±178.2) and 25.0 (±21.1) μg m^–3^, respectively (Figures 2 and 3b).

Following the opening ceremony, no events were held at Alexander Stadium until the athletics events began on August 2nd. During this period, concentrations of PM_10_ and PM_2.5_ returned to levels comparable to the pre-Games period, with average concentrations of 18.5 (±24.8) and 5.3 (±4.4) μg m^–3^, respectively (Figures 2 and 3c). The PM_2.5_/PM_10_ ratio at Alexander Stadium during this period was 0.29, 26% lower than during the pre-event period. PM_2.5_ concentrations at the BAQS and Ladywood urban background sites were 4.3 (±2.2) and 4.1 (±2.0) μg m^–3^, respectively. The PM_2.5_/PM_10_ ratio at Ladywood in this period was 17% lower than in the pre-event period. The decrease of PM_2.5_ concentration to urban background levels when no Games activities were taking place at Alexander Stadium demonstrates that the concentration peaks were linked to the event activities.

Athletics events at Alexander Stadium ran from 2nd to 7th August, with two sessions each day running from 10:00 to 13:30 and 18:30 to 22:00. During this period, particulate concentrations at Alexander Stadium peaked immediately before and after the start and end times of the athletics sessions. The highest concentrations were observed at 18:00, with average concentrations of PM_10_ and PM_2.5_ of 155.6 (±58.7) and 32.9 (±23.4), respectively (Figures 2 and 3c). Average concentrations across the whole athletics period were 68.8 (±68.7) and 14.7 (±13.8) μg m^–3^ for PM_10_ and PM_2.5_, respectively, representing 331% and 139% increases in PM_10_ and PM_2.5_ compared to the pre-Games period. The PM_2.5_/PM_10_ ratio in this period was 0.21 compared to 0.39 during the pre-event period. In contrast, the PM_2.5_/PM_10_ ratio measured at the Ladywood background site was unchanged from the pre-event period at 0.57. Ultrafine particle number concentration peaked during athletics sessions with the number concentration mainly centered in the 20 to 100 nm diameter range (Figure 2). Figure 4 shows a comparison of particle number size distribution between Alexander Stadium and the BAQS urban background site at the start of the evening session (18:00) on 4th August. At Alexander Stadium, the particle number concentration peaked at 33 nm compared to 39 nm at BAQS.

**Figure 4:**
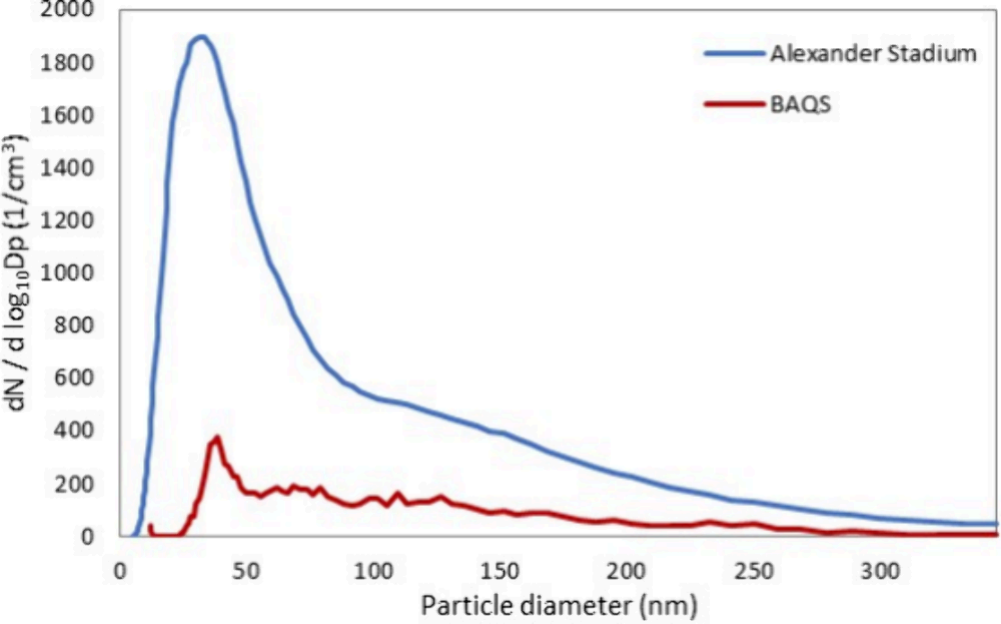
Particle number size distribution at Alexander Stadium (blue) and BAQS (dark red) at the start of the evening athletics session (18:00) on 04 August 2022. This period was selected to coincide with the peak in particulate concentrations, which peaked ahead of the athletics sessions.

The Commonwealth Games closing ceremony was held from 20:00 to 22:00 on August 8th. As with the opening ceremony, the closing ceremony included an extensive fireworks display and catering in the fan zones surrounding the stadium. Particulate concentrations peaked ahead of the ceremony at 19:30, with maximum PM_10_ and PM_2.5_ concentrations of 678.2 and 103.5 μg m^–3^, respectively (Figures 2 and 3d).

Following the closing ceremony, measurements at Alexander Stadium continued until 10th August. On the 9th and 10th of August, vehicles returned to the site, and there was significant activity outside the stadium as the fan areas were dismantled. These activities were directly observed to be generating a large amount of resuspended dust. Average concentrations of PM_10_ and PM_2.5_ following the ceremony were 33.1 (±39.9) and 9.2 (±4.3) μg m^–3^, respectively, with a PM_2.5_/PM_10_ ratio of 0.28. Concentrations on the 9th August peaked between 09:00 and 18:00, suggesting that activity on site during working hours was primarily responsible for the elevated levels.

### PM_2.5_ Composition

In the preathletics period (28th July to 2nd August), when no events were taking place at Alexander Stadium, the organics made up the largest chemical fraction, measured using Q-ACSM, with an average concentration of 5.8 μg m^–3^ (72.3%). SO_4_ (0.9 μg m^–3^), NH_4_ (0.7 μg m^–3^), NO_3_ (0.5 μg m^–3^), and Chl (0.03 μg m^–3^) comprised 11.7%, 8.9%, 6.8%, and 0.3% respectively (Figure 5).

**Figure 5:**
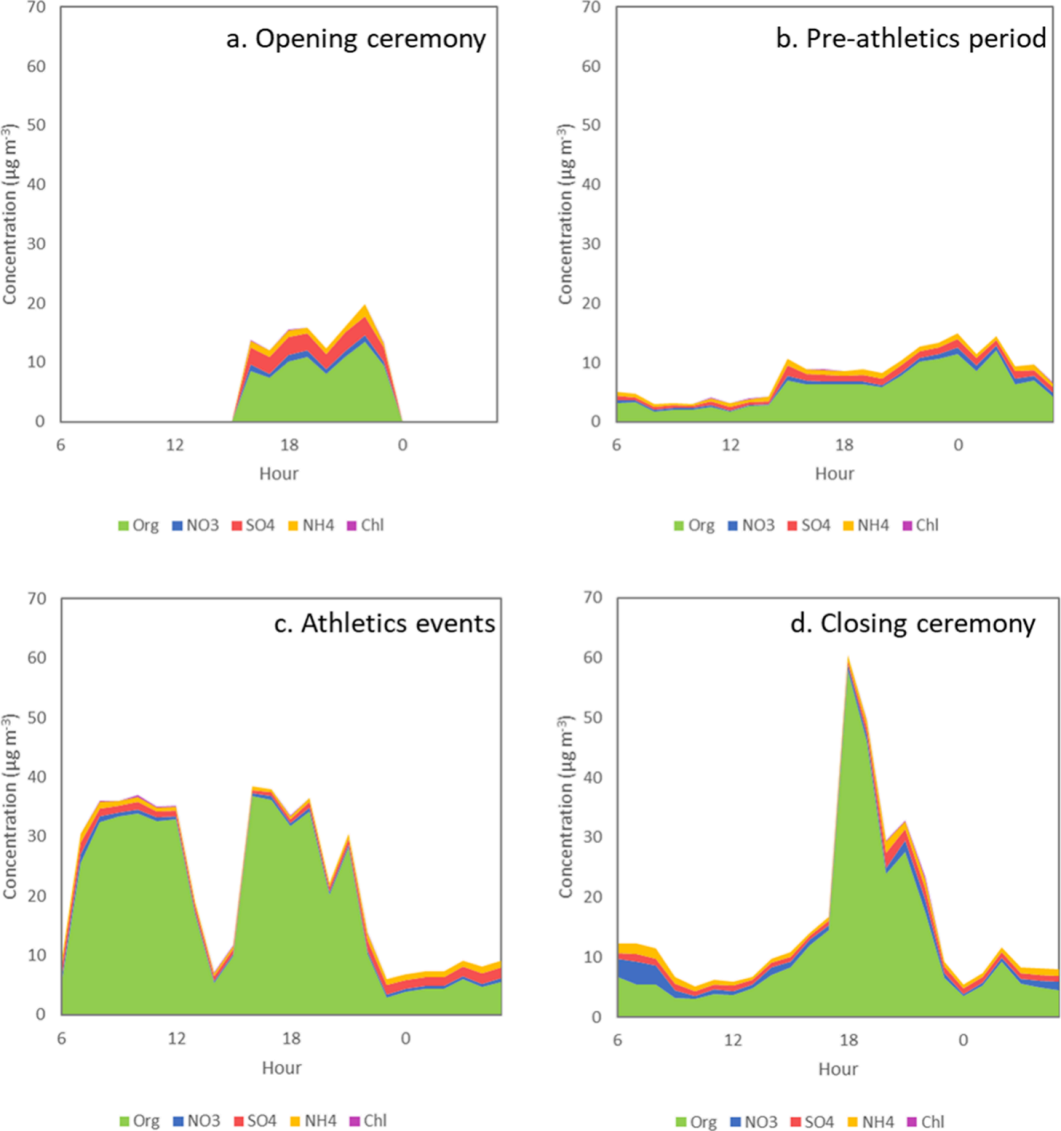
Diurnal profile (6 am–6 am) showing the composition of NR-particulates during the opening ceremony, preathletics period, athletics events, and closing ceremony.

From the 2nd to 7th August, while athletic events were taking place at Alexander Stadium, the organics mass increased to 19.9 μg m^–3^ (88%), dominating the chemical composition of particulates (Figure 5b). This fraction peaked during the athletic sessions in the morning and afternoon, making up 93.8% of the total measured composition (Figure 5c). Also, the organics fraction peaked ahead of the closing ceremony at 18:00 on August 8th, with a maximum hourly average of 57.9 μg m^–3^, making up 95.9% of total measured particulates.

Across the measuring period (29th July to 1st August and 3rd to 6th August), the dominant elements were sulfur (49%), calcium (24%), and iron (12%) (Figure 6). These elements made up 1.4%, 0.7%, and 0.3% of total PM_2.5_, respectively. Sulfur and iron are often associated with fireworks events,^42–44^ and the observed concentrations peaked on the morning of 29th July, following the opening ceremony fireworks display held on 28th July (Figure S5). Potassium and barium, both of which are associated with fireworks activity,^45^ also peaked during this period. However, other elements such as lead, which can also be associated with fireworks, were not elevated. Iron is often also associated with dust and vehicle brake wear.^46–49^ Calcium is associated with crustal and road dust,^43^ which is likely to have been enhanced by construction activity at Alexander Stadium, leaving bare ground and the dry weather during this period.

**Figure 6:**
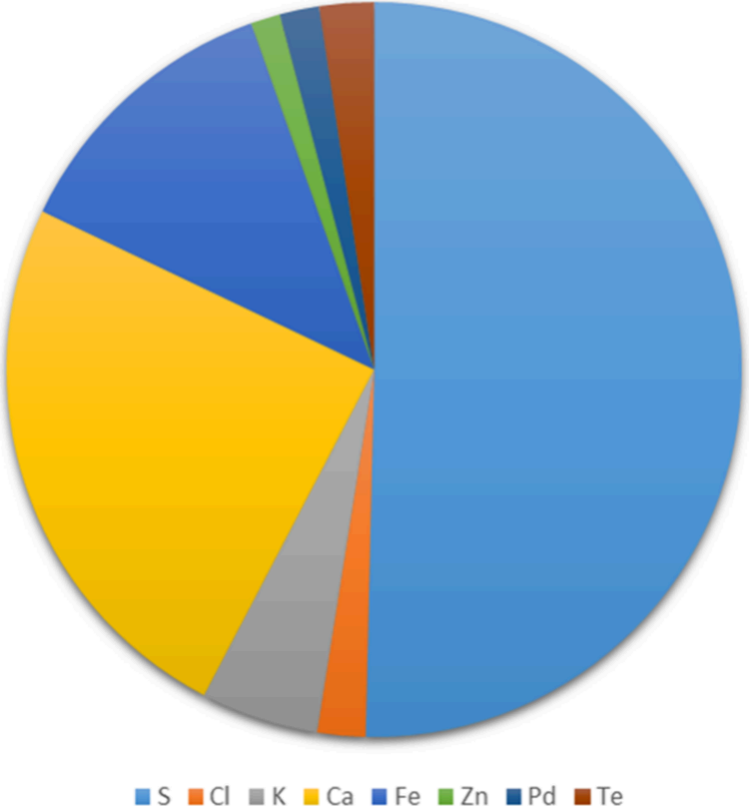
Percentage elemental composition of PM_2.5_ showing elements that constitute more than 1% of measured elemental composition in the periods 29th July to 1st August and 3rd to 6th August when the instrument was operational.

### Sources of Organics in PM_2.5_

Four factors were identified from the PMF analysis (Figure S7) of the Q-ACSM data: hydrocarbon-like organic aerosol (HOA), two cooking-related organic aerosol factors (COA 1 and COA 2), and an oxygenated OA factor (OOA). The HOA mass spectra were dominated by peaks at *m*/*z* 41, 43, 55, and 57. These masses are principally associated with alkanes (C_*n*_H^+^_2*n*–1_ and C_*n*_H^+^_2*n*+1_),^50^ likely from road transport. Despite spectator buses pick up/drop off stations being located away from the stadium on the A34, the time series of HOA concentration follows Games activity, with peaks corresponding to athletics events and the opening and closing ceremonies.

The time series of cooking-related factors shows large peaks at *m*/*z* 41 and 55 and is consistent with cooking factors identified by Allan et al.^51^ and Mohr et al.^52^ A similar cooking factor was also identified by Lanz et al.,^53^ who associated it with charbroiling, which is consistent with much of the cooking activity present at Alexander Stadium (burgers and hot dogs). These factors also showed a high ratio of 55:57 (*m*/*z*), which is a tracer for a COA factor. However, COA-2 had higher signal intensities and explained variation at *m*/*z*’s associated with unsaturated (*m*/*z* 65, 81, 83) and aromatic hydrocarbons (*m*/*z* 77, 91), indicating it to be associated with in-flame charcoal/wood-based cooking.^54^ Meanwhile, COA-1 with higher signals in the lower *m*/*z*’s 27, 29, 41, 43, and 55 and lower aromatic hydrocarbon signals at *m*/*z* 77 and 91 to be associated with cleaner cooking fuels like gas/electric based where majority of OA comes from volatilized/partially oxidized cooking oils rather than incomplete combustion.^55^ The time series of both cooking factors shows peaks corresponding to athletics sessions and the closing ceremony when much of the cooking activity occurred (Figure 7).

**Figure 7:**
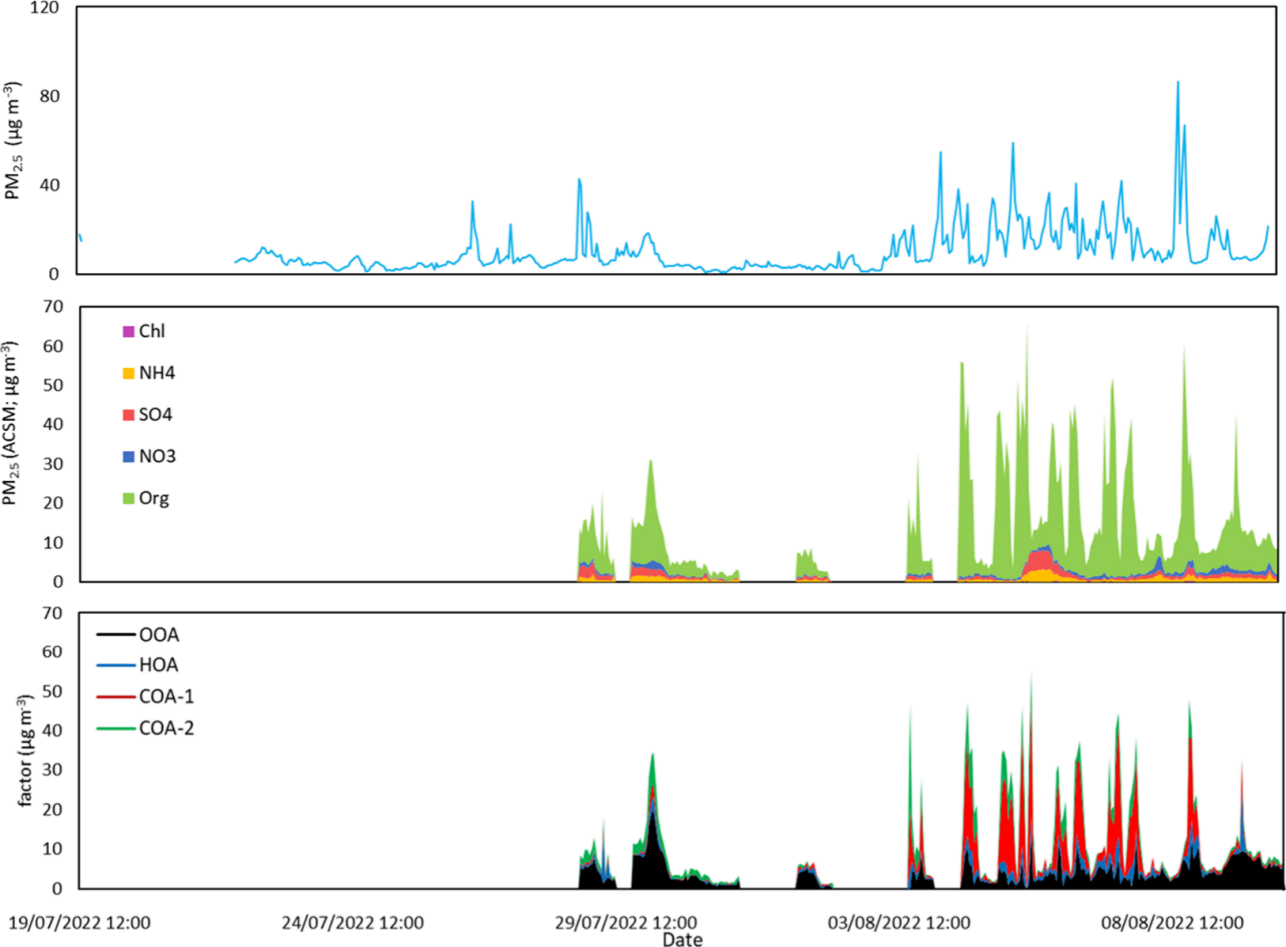
Time series of PM_2.5_ (top), NR-PM_2.5_ composition (middle), and source factors (bottom) at 1 h time resolution.

The OOA shows key peaks at *m*/*z* 29 and 44, consistent with the oxidized organic aerosol fraction identified by Petit et al.^56^ The OOA and COA 1 factors make up the majority of particulate mass, with OOA showing a relatively consistent concentration throughout with a mean of 4.2 (±2.9) μg m^–3^ and COA 1 peaking ahead of the athletics events and opening/closing ceremonies when spectators were purchasing food with a mean of 4.3 (±9.4) μg m^–3^.

## Discussion

### PM_2.5_ Sources and Composition

#### Opening and Closing Ceremonies

The B2022 Games opening and closing ceremonies were held on the evenings of July 27th and August 10th, respectively. The opening ceremony was attended by 30,000 spectators as well as 6600 athletes and 17,000 Games staff and volunteers. The ceremony ran from 20:00 to 22:30 and ended with a large fireworks display. The closing ceremony was also attended by 30,000 spectators as well as staff, volunteers, and performers and ran from 20:00 to 22:00, again ending with a large fireworks display. Spectators arrived at Alexander Stadium by bus ahead of the ceremony’s beginning and were able to shop and eat in the fan zones before entering the stadium.

PM_2.5_ concentrations during the opening and closing ceremonies (27th July and 8th August) were the highest observed at Alexander Stadium during the Games periods, peaking at 88.1 and 103.5 μg m^–3^ on the evenings of the opening and closing ceremonies, respectively. The maximum PM_2.5_ concentrations observed at the BAQS and Ladywood urban background stations on the evening of the closing ceremony (18:00–23:59) were 11.0 μg m^–3^ at BAQS and 9.5 μg m^–3^ at Ladywood, approximately 10 times lower than that at Alexander Stadium.

PM_2.5_ concentrations in the fan areas at both the opening and closing ceremonies peaked between 19:00 and 19:35, ahead of the 20:00 start of the ceremony (Figure 5), when spectators entered the site and spent time in the Fan Zones around the stadium. PM_2.5_ composition between 19:00 and 20:00 ahead of the closing ceremony was dominated by an organic aerosol fraction, with this fraction making up 93% of PM_2.5_ (Figure 5), suggesting that the peak in concentration is being driven by cooking. This is supported by the PMF analysis, which shows both the COA-1 and COA-2 factors peaking between 18:00 and 20:00 immediately ahead of the closing ceremony, accounting for 66% of the source factors (Figure 6). The HOA factor associated with road traffic also peaked at 18:00 ahead of the closing ceremony, suggesting a contribution arising from the bus traffic bringing spectators to the stadium site. The OOA factor peaked before and after the ceremony with the factor peaking an hour later than the peaks in HOA, COA-1, and COA-2, suggesting secondary formation of particles.

PM_2.5_ concentrations decreased during the ceremony and peaked again at the end of the closing ceremony (Figure 5), but at a lower level than before the event ceremony with a maximum PM_2.5_ concentration of 95.2 μg m^–3^ at 22:24. Organic aerosol again dominated the composition but made up a smaller proportion of the total PM_2.5_ concentration (84.3%) than that before the ceremony. The COA-1 and COA-2 factors made up only 16% of the source factors with the OOA and HOA factors dominating. This suggests that the second, postceremony, peak was driven by bus traffic as spectators left the site and the end of the ceremony fireworks display.

#### Emissions from Traffic

A cumulative total of 323,000 spectators attended events at Alexander Stadium across the B2022 Games with a maximum stadium capacity of 32,000 for individual sessions. Access to the Stadium for spectators and volunteers was via public transport and a park-and-ride service with buses stopping on the A34 road to the southwest of the stadium. Only a small number of vehicles connected to the transport of athletes and officials could access the stadium site. During the nonevent period between the opening ceremony and the athletics events beginning, the traffic-associated HOA factor made up 11% of the organic PM_2.5_ source factor at Alexander Stadium, suggesting that the surrounding roads had only a small impact on PM_2.5_ concentrations at the Stadium. This is comparable with other urban background sites in the UK with Crilley et al.,^57^ reporting a 10.2% contribution from traffic at an urban background site in London (North Kensington).

The HOA factor follows the profile of events at Alexander Stadium, demonstrating that transport associated with the B2022 Games impacted particulate concentrations. HOA concentrations peak at the start of athletics sessions with concentrations dropping toward the middle and end of sessions when COA concentrations peak; this is consistent with the peak in traffic around spectator drop off (Figure S8). However, this factor made up only a moderate proportion (16%) of total particulate concentrations during these periods. This suggests that the Games period traffic management was effective in limiting increases in PM_10_ and PM_2.5_ despite increased spectator travel. This is consistent with an analysis of the impact of the B2022 Games on urban background PM_2.5_ and NO_*x*_ concentrations by Liu et al.,^58^ who showed no significant citywide impact on background air pollution during the Games period.

#### Emissions from Cooking

As with the opening and closing ceremonies, large spikes in PM_2.5_ concentrations were observed immediately prior to and after athletics sessions (Figure 3c). Unlike the opening and closing ceremonies, only a small decrease in PM_2.5_ concentration was observed during the athletics sessions. This was consistent with greater movement of spectators between the fan zones and the Stadium to purchase food than during the opening and closing ceremonies.

The composition of NR-PM_2.5_ during the athletic sessions was dominated by organics (Figure 5) with the Org fraction making up 90% of the PM_2.5_ composition during athletic sessions. Ultrafine particle diameter measured by SMPS (Figure 2) peaked in the 20–100 nm range, which is consistent with previous studies investigating cooking emissions.^59^ During the morning and afternoon (7:00–13:00 and 15:00 to 21:00) athletic sessions, the combined contribution of the cooking aerosol factors identified by PMF analysis (COA-1 and COA-2) made up an average of 71% of the total mass. At the same time of day during the preathletics period (29th July–1st August), the combined contribution of the cooking aerosol factors made up 3% of the total source factors. Cooking has previously been identified as a significant source of particulates in football stadiums^21^ and during festivals.^60^ In addition to cooking, both studies identified cigarette smoke as a large source of particulate matter. At Alexander Stadium, smoking was prohibited within venue perimeters, so a signal from cigarette smoke was not expected.

This cooking-related PM_2.5_ was the main driver of peak pollution at the Alexander Stadium. Cooking has previously been shown to be a significant source of PM_2.5_ in urban environments,^61,62^ with emissions dependent on the type of cooking.^63^ The concession stands present at Alexander Stadium were typical of those at many event venues, focusing on fast food, particularly burgers, hot dogs, and stir-fried dishes. Meat grilling^64^ and frying^65^ have been shown to emit high levels of PM_2.5_. The large influence of cooking sources on the peak PM_2.5_ concentrations at Alexander Stadium during the B2022 Games was therefore likely driven by the cuisines on offer and the amount of food prepared on site.

### Exposure to PM_2.5_

The high concentrations of particulates at Alexander Stadium during the B2022 Games period mean that for spectators attending the stadium, the visit results in a large increase in their daily particulate exposure. For a spectator attending a single session (17:00–23:00), daily exposure to PM_10_ and PM_2.5_ would be increased by 207% and 73%, respectively, relative to Birmingham urban background concentrations. For a spectator attending a full day of athletic events (10:00–23:00), their daily exposure to PM_10_ and PM_2.5_ would be increased by 360% and 125%, respectively. While the increase in spectator exposure to particulates is significant, it is likely limited to a single session or day. For a member of the Games staff or a volunteer working every day at the stadium, this exposure could be repeated over the 7 days of events. The average particulate concentrations to which they were exposed during this period are 17 μg m^–3^ for PM_2.5_ and 188 μg m^–3^ for PM_10_, compared to just 4 μg m^–3^ for PM_2.5_ and 9 μg m^–3^ for PM_10_ if they had been exposed only to the Birmingham urban background during this period.

The WHO provide air quality guidelines recommending limit values for air pollutant concentrations in order to achieve air quality that protects public health.^66^ The WHO 24h guideline concentrations are 45 and 15 μg m^–3^ for PM_10_ and PM_2.5_, respectively, with 3–4 exceedances allowed per year (99th percentile). A spectator, staff member, or volunteer attending all the athletic events at Alexander Stadium would experience air quality exceeding these guidelines. Morawska et al.^67^ recommend that the WHO 24-h air quality guidance level for PM_2.5_ (15 μg m^–3^) be adopted for public buildings but with a 1-h averaging time. For staff working in the catering stands, close to the main emission sources of PM_2.5_ on site, this recommendation will be greatly exceeded. This exposure will be further increased for staff members working in the concession stands as they may attend (or work at) multiple events each year.

### Implications

Air pollutant concentrations at event venues depend on event-related emission sources, regional background concentrations, weather, ventilation, and other factors. The relative impact of PM_2.5_ emissions from catering and other sources at venues will, therefore, depend on the regional background. For events held in cities with comparatively high urban background PM_2.5_ concentrations, for example, the Beijing Olympics (58.5 μg m^–3^)^16^ or the Delhi Commonwealth Games (60–80 μg m^–3^),^13^ the relative impact of on-site emissions of PM_2.5_ may be comparatively low, but they nonetheless impact absolute emissions and therefore exposure. In cities such as Birmingham where background concentrations are lower (but still significantly higher than the 5 μg m^–3^ WHO guideline level) emissions from catering and other sources will significantly increase PM_2.5_ concentrations at the venue.

While events on the scale of the Commonwealth and Olympic Games are relatively infrequent, large sporting events are common in most major cities. In the UK alone, there are 34 venues with a capacity of greater than 30,000, making them comparable or larger than Alexander Stadium during the B2022 Games. In addition to sporting events, there are also numerous music and cultural festivals, with 6.5 million people attending music festivals in the UK in 2022.^68^ In order to cater for large numbers of attendees, these events often host numerous cooking sources similar to those observed at Alexander Stadium. Members of the public attending these events are likely to experience a significant increase in their short-term exposure to PM_2.5_, and staff and performers regularly attending events will increase their long-term exposure to PM_2.5_.

## Conclusions

The B2022 Games fell between two periods of relatively high pollution in mid-July and mid-August, associated with stagnant air masses during the early July and August heat waves. During the Games, background air quality in Birmingham was good, with PM_10_ and PM_2.5_ concentrations averaging 9.3 (±4.3) and 4.5 (±2.4) μg m^–3^, respectively, at the Birmingham Ladywood site.

The concentration and composition of PM_2.5_ were measured in a fan area adjacent to but outside Alexander Stadium in Birmingham. PM_2.5_ concentrations at Alexander Stadium during the event period averaged 17 μg m^–3^ with concentrations peaking ahead of the athletics events and opening and closing ceremonies. PM_2.5_ concentrations peaked at 103.5 μg m^–3^ before the closing ceremony, approximately 10 times higher than that at nearby urban background monitoring stations. Concentrations of PM_10_ also peaked during the athletics period, as well as the opening and closing ceremonies, with PM_10_ concentrations averaging 69 μg m^–3^ during the athletics period and peaking at 696 μg m^–3^ during the opening ceremony.

The high levels of PM_2.5_ during event periods were shown to be driven primarily by cooking sources. During the athletic sessions and closing ceremony, when PM_2.5_ concentrations peaked, the NR-PM_2.5_ composition was dominated by organics. PMF analysis of the organic particle matter fraction identified 4 factors: HOA, COA-1, COA-2, and OOA. During athletic sessions, the cooking aerosol factors identified by PMF analysis (COA-1 and COA-2) made up 71% of the total source factors. Although fireworks were used during the opening and closing ceremonies and before the athletics session, the observed increase in PM_2.5_ in the fan areas surrounding the stadium was predominantly driven by these cooking sources.

While the impact of fireworks on air quality at major cultural and religious festivals is well reported,^28,69^ with studies demonstrating a large increase in particulate concentrations driven by fireworks, the impact of event catering on air quality during major events has received less focus. This study demonstrates the importance of cooking as a source of PM_2.5_ during major events. This cooking generated PM_2.5_ results in increased exposure for spectators attending events and particularly for staff who attend multiple events.

## Supplementary Material



## Data Availability

Data from the Birmingham Ladywood AURN station were made available under the Open Government Licence: Crown 2025 copyright Defra via uk-air.defra.gov.uk, licensed under the Open Government Licence (OGL). Map data were from OpenStreetMap under the Open Database License (https://www.openstreetmap.org/copyright).

## Notes

Supporting Information contains a comparison between total mass recorded on Fidas and ACSM (S1); meteorological information (S2 and S3); back trajectory analysis (S4); elemental concentration time series of selected elements (S5); and supporting figures for PMF solution (S6 – S8) (PDF)

## †

Center for Study of Science, Technology and Policy (CSTEP), Bangalore, KA 560094, India (V.L.)
