# Supplementary material for: The Impact of Hospitality on Air Quality at a Major Sporting Event
Source: ACS EST Air. 2026 Feb 2;3(2):279–90. doi: 10.1021/acsestair.5c00142 (PMC12910594; doi:10.1021/acsestair.5c00142)
Supplement: Supplementary file 1 [file ea5c00142_si_001.pdf]

The impact of hospitality on air quality at a major sporting event: Supplementary Information

W. Joe F. Acton[a *], Vipul Lalchandani[a,†], Mao Du[a], Siqi Hou[a], Deepchandra Srivastava[a], Zongbo Shi[a], William J. Bloss[a]

a) *School of Geography, Earth and Environmental Sciences, University of Birmingham, Birmingham, B15 2TT, UK*

† now at Center for Study of Science, Technology and Policy (CSTEP), Bangalore, KA 560094, India

**Email: w.j.f.acton@bham.ac.uk*

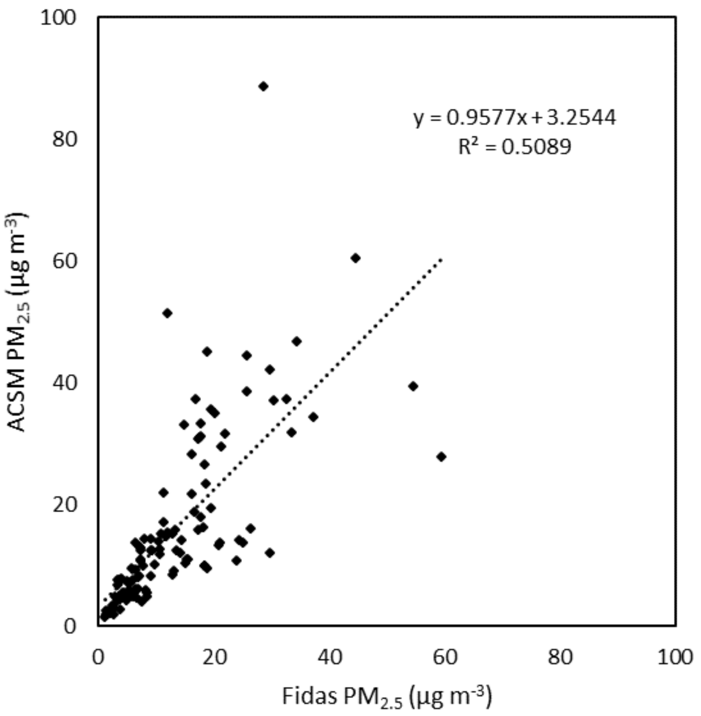

Figure S1, comparison between the total mass of $PM_{2.5}$ recorded on Fidas and ACSM

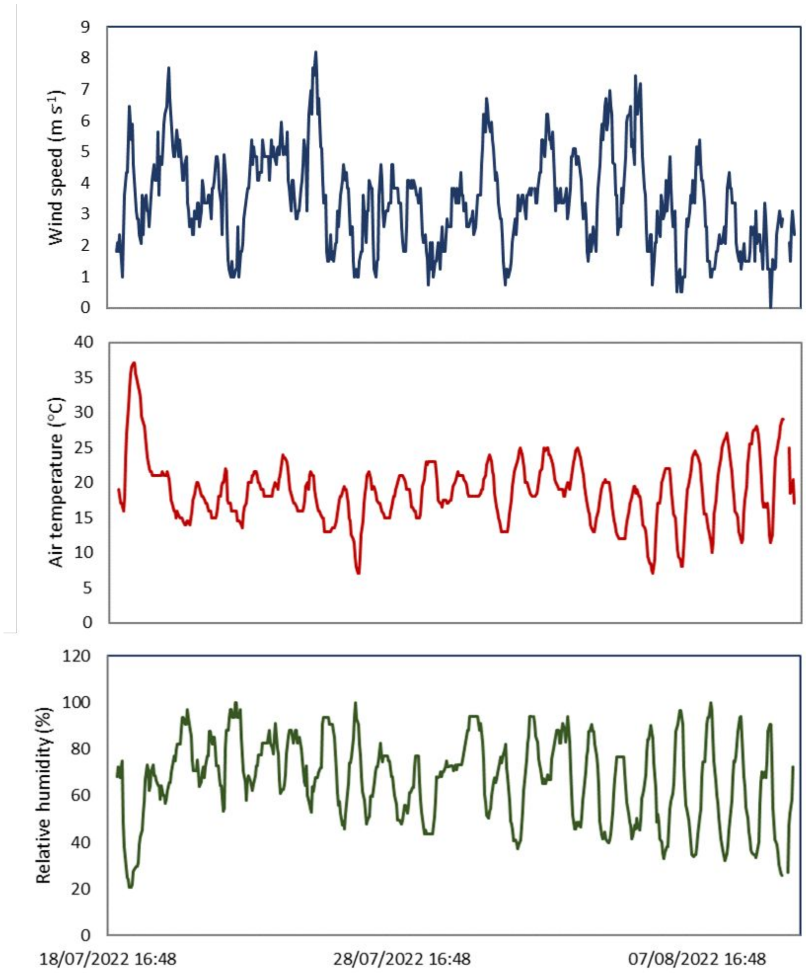

Figure S2: Windspeed (top), air temperature (middle) and relative humidity (bottom) at BAQS during the B2022 Commonwealth Games

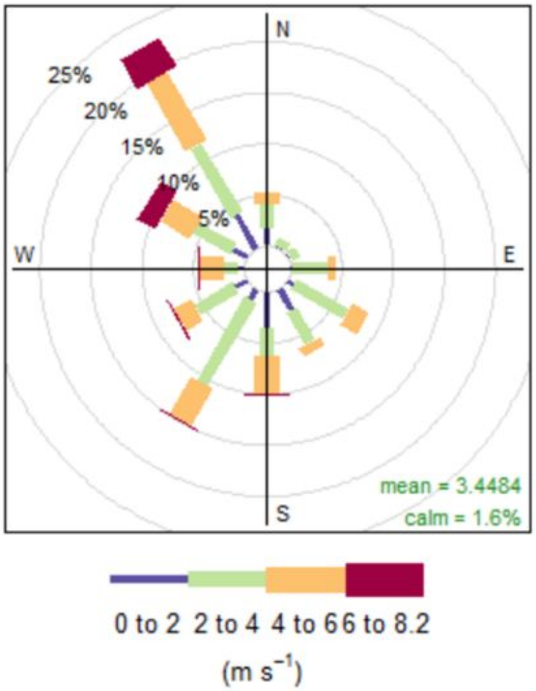

Figure S3: Windrose showing windspeed and direction recorded at BAQS from 19/07/2022 to 10/08/2022.

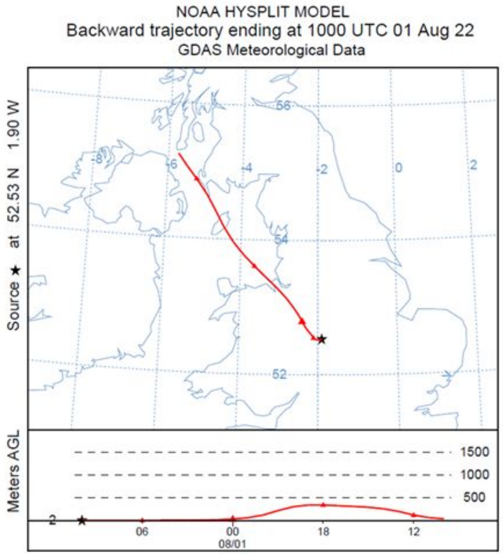
NOAA HYSPLIT MODEL
Backward trajectory ending at 1000 UTC 01 Aug 22
GDAS Meteorological Data
Source ★ at 52.53 N 1.90 W
Meters AGL
1500
1000
500
06
00
18
12
08/01

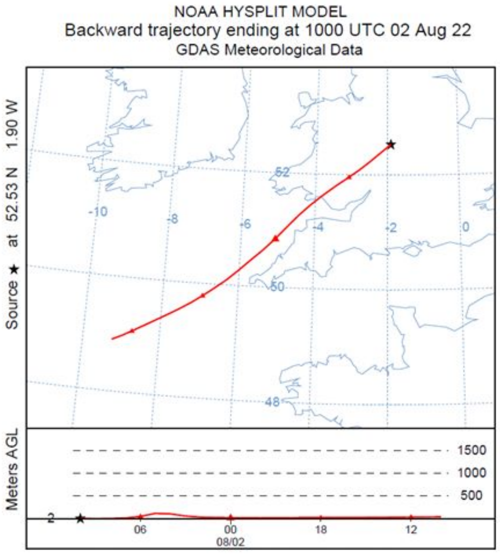
NOAA HYSPLIT MODEL
Backward trajectory ending at 1000 UTC 02 Aug 22
GDAS Meteorological Data
Source ★ at 52.53 N 1.90 W
Meters AGL
1500
1000
500
06
00
18
12
08/02

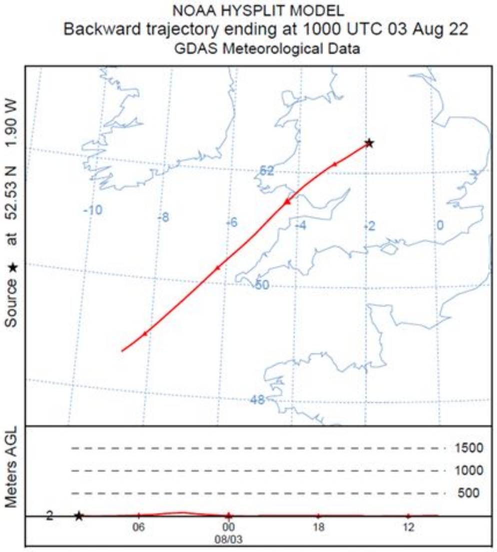
NOAA HYSPLIT MODEL
Backward trajectory ending at 1000 UTC 03 Aug 22
GDAS Meteorological Data
Source ★ at 52.53 N 1.90 W
Meters AGL
1500
1000
500
06
00
18
12
08/03

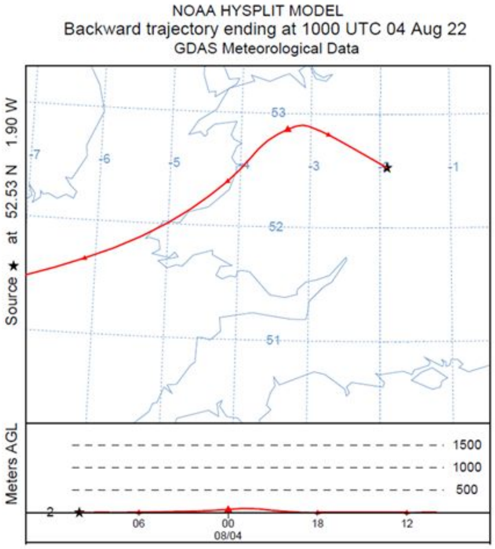
NOAA HYSPLIT MODEL
Backward trajectory ending at 1000 UTC 04 Aug 22
GDAS Meteorological Data
Source ★ at 52.53 N 1.90 W
Meters AGL
1500
1000
500
06
00
18
12
08/04

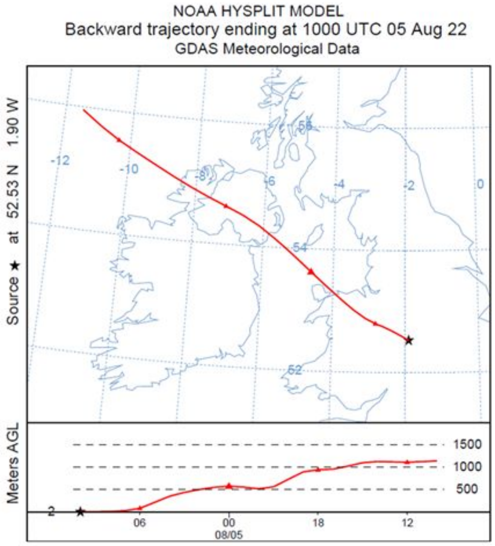
NOAA HYSPLIT MODEL
Backward trajectory ending at 1000 UTC 05 Aug 22
GDAS Meteorological Data
Source ★ at 52.53 N 1.90 W
Meters AGL
1500
1000
500
06
00
18
12
08/05

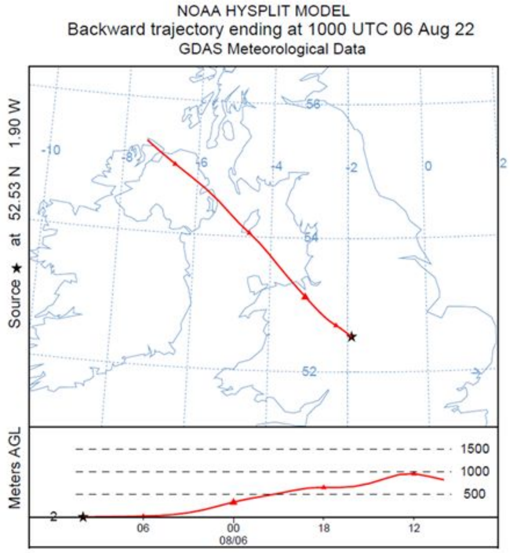
NOAA HYSPLIT MODEL
Backward trajectory ending at 1000 UTC 06 Aug 22
GDAS Meteorological Data
Source ★ at 52.53 N 1.90 W
Meters AGL
1500
1000
500
06
00
18
12
08/06

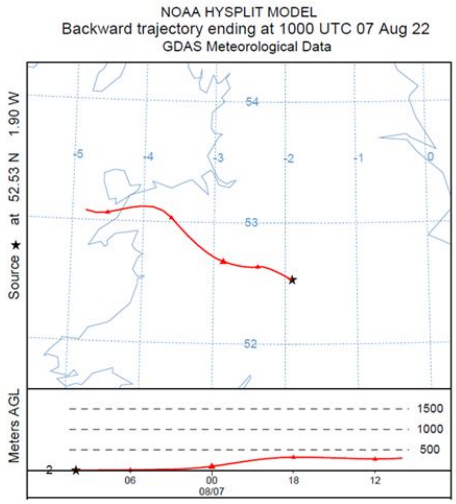

Figure S4. Back trajectory analysis using the HYSPLIT transport and dispersion model (Rolph et al., 2017; Stein et al., 2015) showing 24h trajectories ending at 10:00 for athletics period (1st-7th August 2022)

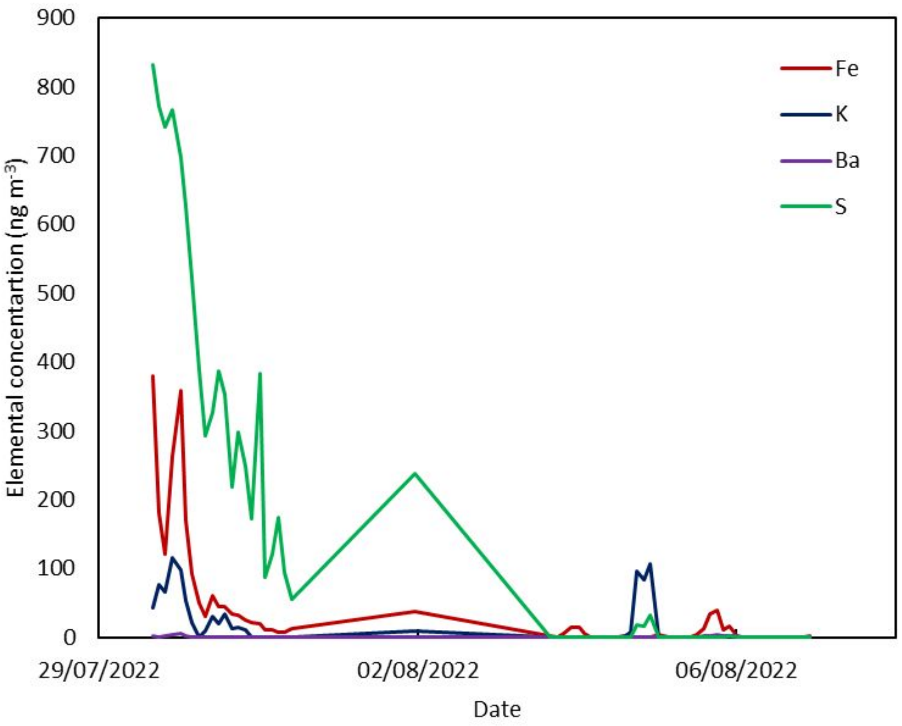

Figure S5. Elemental concentration of selected elements (Fe, K, Ba and S) recorded at Alexander Stadium

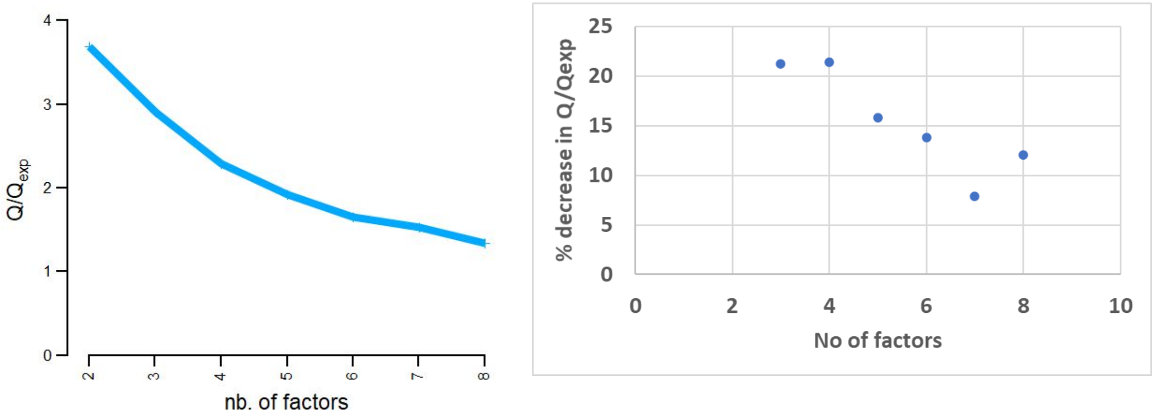

Figure S6. Q/Qexp vs number of factors and percent decrease in Q/Qexp vs number of factors in the PMF solution.

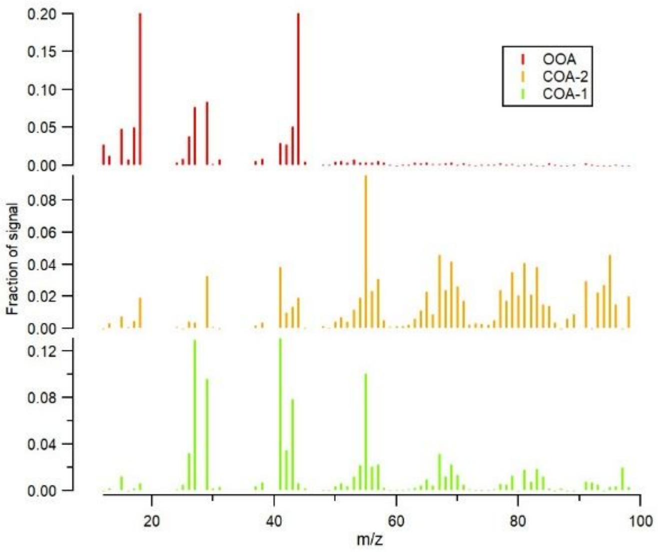

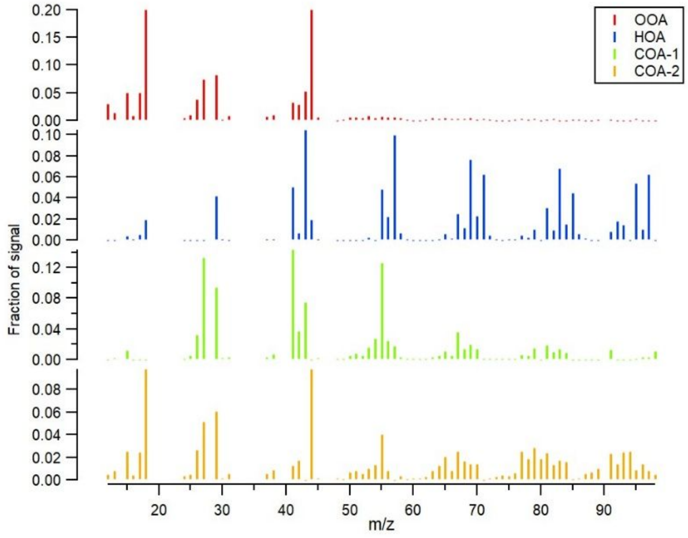

Figure S7. Factor profiles of 3-factor and 4-factor PMF solution

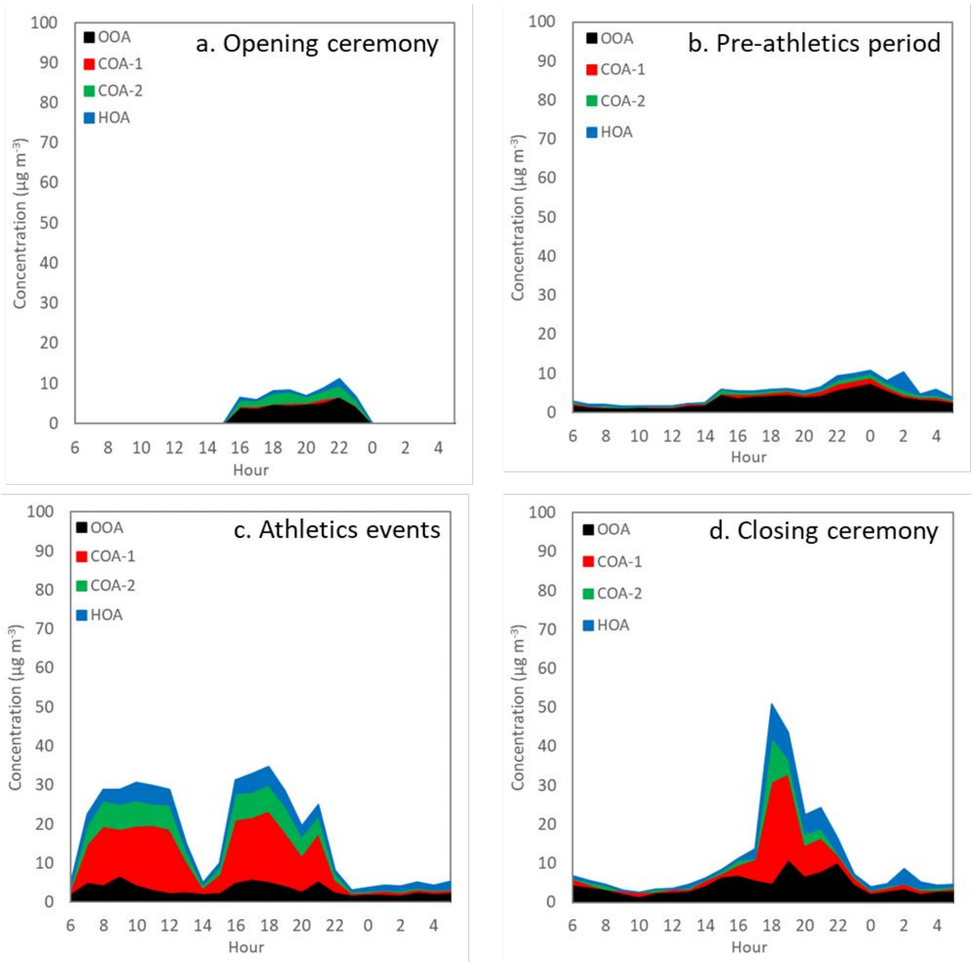

Figure S8 Diurnal profile (6 am – 6 am) of the 4 PMF solution factors during the opening ceremony, pre-athletics period, athletics events and closing ceremony.

References

Rolph G., Stein A., and Stunder B.: Real-time Environmental Applications and Display sYstem: READY. Environmental Modelling & Software, 95, 210-228, 2017 https://doi.org/10.1016/j.envsoft.2017.06.025

Stein A.F., Draxler R.R, Rolph G.D., Stunder B.J.B., Cohen M.D., and Ngan F.: NOAA's HYSPLIT atmospheric transport and dispersion modeling system, Bull. Amer. Meteor. Soc., 96, 2059-2077, 2015. http://dx.doi.org/10.1175/BAMS-D-14-00110.1
